# In B-CLL, the codon 72 polymorphic variants of p53 are not related to drug resistance and disease prognosis

**DOI:** 10.1186/1471-2407-5-105

**Published:** 2005-08-18

**Authors:** Isrid Sturm, Andrew G Bosanquet, Michael Hummel, Bernd Dörken, Peter T Daniel

**Affiliations:** 1 Department of Hematology, Oncology and Tumor Immunology, University Medical Center Charité, Campus Berlin-Buch and Department of Hematology and Oncology, University Medical Center Charité, Campus Virchow Klinikum, Humboldt University, 13353 Berlin, Germany; 2 Clinical and Molecular Oncology, Max Delbrück Center for Molecular Medicine, 13125 Berlin, Germany; 3 Bath Cancer Research, Royal United Hospital, Bath, BA1 3NG, UK; 4 Institute of Pathology, University Medical Center Charité, Campus Benjamin Franklin, Humboldt University, 12300 Berlin, Germany

## Abstract

**Background:**

A common sequence polymorphism at codon 72 of the p53 gene encoding either arginine or proline was recently shown to be functionally relevant for apoptosis induction in vitro. In B-type chronic lymphocytic leukemia (B-CLL), p53 gene mutations occur in a subset of patients and are associated with impaired survival and drug resistance. Here, we address the functional relevance of the codon 72 single nucleotide (SNP) polymorphism for cell death sensitivity following exposure to clinically employed cytotoxic drugs and γ-irradiation.

**Methods:**

138 B-CLL samples were analysed by SSCP-PCR and sequencing for single nucleotide polymorphism at codon 72 of the p53 gene. The in vitro cytotoxicity assay (DiSC-assay) was performed with 7 drugs (chlorambucil, mafosfamide, fludarabine phosphate, methylprednisolone, doxorubicin, vincristine) or γ-irradiation.

**Results:**

Of the138 B-CLL samples, 9 samples were homozygous for proline (Pro/Pro), 78 samples homozygous for arginine (Arg/Arg), and 49 samples heterozygous (Arg/Pro). No differences were found for patient survival and cell death triggered by 7 cytotoxic drugs or γ-irradiation.

**Conclusion:**

These data indicate that polymorphic variants of p53 codon 72 are not clinically relevant for apoptosis induction or patient survival in B-CLL.

## Background

The tumor suppressor gene p53 plays a central role in the induction of cell cycle arrest, senescence and apoptosis [1-5]. The polyproline domain (PP domain) of p53 spanning amino acids 62–91 is involved in apoptosis induction and facilitates transactivation of pro-apoptotic genes by p53 [6].

Located in this PP domain is at codon 72 a common single nucleotide polymorphism (SNP), resulting in either a proline residue (p53Pro) or an arginine residue (p53Arg). Thus, each individual inherits a p53 genotype that can be heterozygous (Arg/Pro) or homozygous for either arginine (Arg/Arg) or proline (Pro/Pro). The polymorphism is balanced, varies with latitude and race, and is maintained at different allelic frequencies across the population [7]. These two SNPs appear to be different both biochemically and biologically [8-11]. Differences in apoptosis susceptibility to cytotoxic drugs were described [12,13], and the response and survival to radiochemotherapy in clinical samples of squamous cell carcinomas was found to be increased in case the arginine allele is retained [13].

Chronic lymphocytic B-cell lymphoma is still an incurable disease and may be addressed as a disease of intrinsic apoptosis deficiency. It is a disease where the mutational status of the p53 gene is linked to patient survival [14] (and references herein). We therefore asked whether the codon 72 polymorphism is of clinical relevance for in vitro resistance to cytotoxic drugs, γ-irradiation and patient prognosis.

## Methods

### Patients

Samples from 138 B-CLL patients (99 male, 39 female, age 63.2 ± 0.5 (mean ± SEM) were analysed. Peripheral blood was analysed for drug sensitivities using fresh cells. The same samples were analysed for mutations in the p53 DNA binding domain and of the p53 codon 72 SNP, using snap frozen cells from the same specimens. Only patients with high peripheral blood leukocyte count were included in this analysis (median WBC 120.8/nl, range 20.7–1262.2/nl). The Binet stage was A in 29 cases, B in 24 cases, A/B in 15 cases (due to insufficient information on clinical lymph node status) and C in 62. Of the 138 patients, 80 were pretreated (58%) with one to six drug regimens (mean number of pretreatments for these 80 patients ± SEM: 1.93 ± 1.005). Median survival was 30.1 months; median follow up for the 17 censored patients was 97.9 months. This study was performed in accordance with local ethical standards and the declaration of Helsinki.

### Analysis of p53 codon 72 polymorphism

SNP in the p53 codon 72 were analysed by genomic SSCP-PCR analysis and DNA sequencing. The primers were CGG ACG ATA TTG AAC AAT GG (sense) and CGT TTT CTG GGA AGG GAG AG (antisense), resulting in a PCR product of 167 bp. A standard PCR reaction in 50 μl with a final concentration of 0.6 mM MgCl_2 _was performed (40 cycles, annealing temperature 56°C). PCR products were denatured and separated on a nondenaturating 10% polyacrylamide gel at 500 V and 50 mA for 2 h at 22°C and analysed by silver staining [15,16]. Sequence polymorphism was confirmed by DNA sequencing, as described [14]. A result was obtained in 136 samples (98.6%). Mutations in the DNA-binding domain of the p53 gene (exon 5 to 8) has been analyzed previously by the use of SSCP-PCR and sequencing as described in detail elsewhere [14].

### Chemosensitivity assay

B-CLL cells were exposed to cytotoxic drugs (chlorambucil, mafosfamide, fludarabine phosphate, methylprednisolone, doxorubicin, vincristine) or γ-irradiation (2 Gy) and cultured for 92 h. Percentages of drug-induced cell death and LD90 doses were determined by the use of a standardized morphometric test, the DiSC assay [17,18].

### Data analysis

For intervariable assessment, the non-parametric Mann-Whitney U-test (for 2 groups) or the Kruskal-Wallis test (for 3 groups) or the χ^2^-test or Fisher's exact test for categorical parameters were applied. Overall survival was estimated by the Kaplan-Meier product-limit method. For the chemosensitivity data, LC_90 _doses were determined by calculating the log dose at which the fitted survival probability was equal to 0.1. LC_90 _values were logged (base 10) before calculation of mean and SEM, as described [19].

## Results

### Codon 72 SNP and clinical data

Of the 138 samples analysed, 136 could be amplified by genomic PCR : 78 samples were Arg/Arg, 9 samples were Pro/Pro, and 49 samples were Arg/Pro. Patients age and gender was not different in the subgroups (63.3 ± 1.07 years; male/female 55/23 in Arg/Arg, 65.2 ± 2.7 years, male/female 6/3 in Pro/Pro, 63.2 ± 1.5; male/female 37/12 in Pro/Arg). In the Binet's stage, the distribution was: stage A: 18 Arg/Arg, 1 Pro/Pro, 10 Pro/Arg; stage B: 10 Arg/Arg, 1 Pro/Pro, 13 Pro/Arg; stage C: 37 Arg/Arg, 6 Pro/Pro, 18 Pro/Arg; stage A or B: 9 Arg/Arg, 1 Pro/Pro, 5 Pro/Arg (p = 0.6).

78 patients were pretreated with a median of 2 treatment regimens. There was, however, no association between pretreatment status and codon 72 SNP (p = 0.6).

### Codon 72 SNP and IgVH and p53 gene mutational analysis

In 113 samples, the IgV_H_-gene status was known, and in all 136 cases the p53 genotype was known [14]. Concerning the IgV_H_-gene status, of the hypermutated samples, 19 were Arg/Arg, 0 Pro/Pro, 10 Pro/Arg and of the pre-germinal center samples, 50 were Arg/Arg, 6 Pro/Pro, 28 Pro/Arg (p = 0.3). Concerning the p53 genotype of the B-CLL samples, of the 22 p53-mutated samples, 17 were Arg/Arg, 2 Pro/Pro, 3 Pro/Arg and of the 114 p53-wildtype samples, 61 were Arg/Arg, 7 Pro/Pro, 46 Pro/Arg (p = 0.06).

### Codon 72 SNP and survival

**Figure 1:**
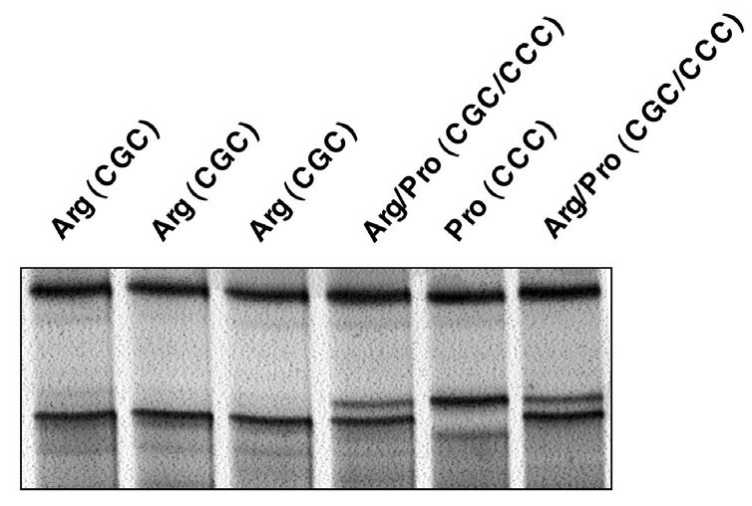
**Analysis of p53 codon 72 SNP in B-CLL**. Silver-stained polyacrylamide gel of 6 B-CLL samples analysed by SSCP-PCR for the codon 72 SNP. Sequencing of the 138 B-CLL samples confirmed the polymorphisms in p53 gene, codon 72 (exon 4): 6.6% of the samples were homozygous for proline (Pro/Pro), 57.4% were homozygous for arginine (Arg/Arg), and 36% were heterozygous with both an arginine and a proline allele (Arg/Pro).

There was no difference in overall survival for the 3 different groups (p = 0.885) (Fig. 1B). This was also true when only the p53 wild type samples were analysed (p = 0.46).

### Codon 72 SNP and cell death sensitivity

For none of the tested drugs (chlorambucil, mafosfamide, doxorubicine, vincristine, fludarabine, caldribine and methylprednisolone), a correlation to the p53 codon 72 SNP was seen. This was the case irrespective of the p53 mutational status (Table 1). For γ-irradiation, there was a trend (p = 0.099) for better survival of the B-CLL cells when the sample is homozygous for proline. This finding is in accordance with a recent report on response rates and survival of head and neck carcinoma patients treated with radiochemotherapy where patients with a homozygous proline genotype had an impaired outcome to treatment [13]. This association of the Pro/Pro genotype failed, however, to attain statistical significance. This, together with the lack of correlation with patient survival indicates a rather limited clinical relevance of such codon 72 polymorphisms.

## Discussion

Recently, a sequence polymorphism at codon 72 the p53 gene (exon 4) encoding either arginine (CGC) or proline (CCC) was suggested to result in a drastically altered biological and biochemical behaviour of p53 *in vitro*. Compared to the proline encoding allele, the arginine allele appeared to trigger a more pronounced apoptosis response, whereas the proline allele induced significantly more G1 arrest [8,10,13].

The question of clinical relevance was addressed in a few case control studies. There, the proline allele was associated with urothelial [20], thyroid [21,22], and colorectal carcinomas [23] and chronic myeloid leukemia [24], whereas homozygosity for arginine was associated with advanced lung cancer [25]. Concerning patient outcome, in Italian breast cancer patients, the retention of an arginine allele was correlated with a reduction of survival in one study [26]. Another study addressed the clinical relevance of this SNP for treatment response in solid tumors. There, the homozygous proline genotype was correlated with impaired response to radiochemotherapy and reduced survival in head and neck carcinoma [13]. Furthermore, p53 protein encoded by the arginine allele appears to be more susceptible to HPV-E6 protein-induced degradation [27]. Although many studies investigated the relationship of the allelic distribution and susceptibility to HPV-associated cervical carcinoma, two recent meta-analyses could not establish such a correlation with the p53 gene codon 72 SNP [28,29].

In B-CLL, the p53 gene is known to be of clinical relevance concerning survival and treatment response [14]. We therefore investigated the potential clinical relevance of the p53 gene codon 72 SNP in a cohort of 138 samples from patients with B-CLL with respect to survival and drug sensitivity.

The distribution of the proline and arginine alleles in the B-CLL samples was, however, not different of frequency distributions observed in the general population [7]. Moreover, we found no correlation between codon 72 p53 SNP and p53 Exon 5–8 mutations or the IgV_H _hypermutation status or clinical Binet stage. Likewise, survival was not different in the p53 codon 72 SNP subgroups. Furthermore, no correlation with in vitro drug sensitivities was seen. The trend for reduced sensitivity to irradiation in case of homozygosity for proline is in accordance with a previous report in head and neck cancer [7], but fails to reach statistical significance. These data indicate that the codon 72 SNPs of p53 have per se no clinical relevance, at least in B-CLL. This may indicate either that B-CLL differs significantly from other tumors with regard to the regulation of cell death induced by cytotoxic anticancer therapies or that positive reports from other studies in clinical samples are false positive, eg. due to small sample size.

## Conclusion

Although p53 gene mutations in the DNA-binding domain are clinically relevant for patients with B-CLL, the p53 gene codon 72 SNP was not found to be of clinical relevance in patients with B-CLL, neither for patient survival nor for *ex vivo *sensitivity for cytotoxic drugs and γ-irradiation.

## Competing interests

The author(s) declare that they have no competing interests.

## Authors' contributions

IS compiled the study, carried out the p53 genetic analysis, performed the statistical analysis and drafted the manuscript. AB carried out the cell death assays and provided the clinical samples and data, MH analysed the Ig_H _mutational status, BD participated in the design of the study, PTD participated in the design of the study and drafted the manuscript. All authors read and approved the final manuscript.

## Pre-publication history

The pre-publication history for this paper can be accessed here:



## Figures and Tables

**Figure 2:**
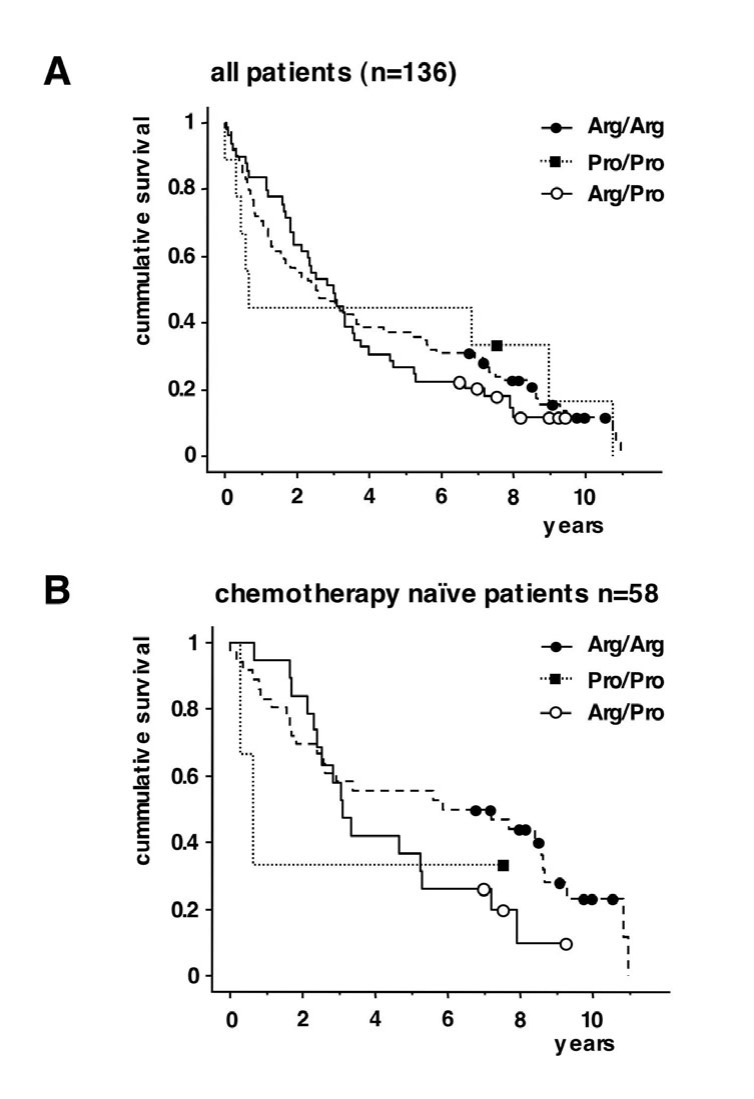
**Kaplan-Meier-survival estimates for overall survival for B-CLL patients with an Arg/Arg, Pro/Pro and Arg/Pro p53 codon 72 genotype**. **A**: All patients. Squares and circles indicate censored patients. Log rang test p = 0.9 **B**: Subgroup of chemotherapy naïve patients ("no pretreatment") (n = 58). Squares and circles indicate censored patients. Log rang test p = 0.3.

**Table 1:** Resistance to ionising irradiation and cytotoxic drugs in relation to the p53 codon 72 status

	**all patients**				**p53 wild type**			
				
	**p53 codon 72 Arg/Arg**	**p53 codon 72 Pro/Pro**	**p53 codon 72 Arg/Pro**	**p**	**p53 codon 72 Arg/Arg**	**p53 codon 72 Pro/Pro**	**p53 codon 72 Arg/Pro**	**p**
γ-irradiation (% surviving cells)	36.3 ± 4.7 (n = 49)	50.0 ± 10.1 (n = 7)	24.2 ± 4.8 (n = 26)	0.099	30.6 ± 4.8 (n = 39)	54.7 ± 13.4 (n = 5)	22.9 ± 4.9 (n = 25)	0.14
log_10 _LC_90 _chlorambucil	0.73 ± 0.07 (n = 78)	0.76 ± 0.19 (n = 9)	0.62 ± 0.08 (n = 49)	0.63	0.6 ± 0.08 (n = 61)	0.76 ± 0.22 (n = 7)	0.57 ± 0.08 (n = 46)	0.70
log_10 _LC_90 _mafosfamide	0.32 ± 0.05 (n = 75)	0.37 ± 0.12 (n = 9)	0.21 ± 0.05 (n = 47)	0.39	0.3 ± 0.06 (n = 59)	0.38 ± 0.16 (n = 7)	0.21 ± 0.05 (n = 44)	0.42
log_10 _LC_90 _fludarabine	0.18 ± 0.08 (n = 78)	0.13 ± 0.18 (n = 9)	-0.32 ± 0.1 (n = 49)	0.32	0.11 ± 0.1 (n = 61)	0.22 ± 0.23 (n = 7)	-0.06 ± 0.01 (n = 46)	0.28
log_10 _LC_90 _cladribine	-0.76 ± 0.1 (n = 78)	-0.85 ± 0.18 (n = 9)	-1.0 ± 0.11 (n = 49)	0.10	-0.89 ± 0.1 (n = 61)	-0.79 ± 0.22 (n = 7)	-1.04 ± 0.11 (n = 46)	0.22
log_10 _LC_90 _vincristine	0.13 ± 0.1 (n = 55)	0.01 ± 0.27 (n = 8)	-0.22 ± 0.11 (n = 39)	0.71	-0.14 ± 0.11 (n = 40)	0.05 ± 0.33 (n = 6)	-0.25 ± 0.11 (n = 36)	0.59
log_10 _LC_90 _doxorubicin	-0.55 ± 0.04 (n = 78)	-0.52 ± 0.06 (n = 9)	-0.61 ± 0.05 (n = 49)	0.64	-0.55 ± 0.05 (n = 61)	-0.49 ± 0.07 (n = 7)	-0.63 ± 0.05 (n = 46)	0.35
log_10 _LC_90 _methylprednisolone	0.99 ± 0.14 (n = 78)	1.36 ± 0.45 (n = 9)	0.95 ± 0.18 (n = 48)	0.70	0.99 ± 0.16 (n = 61)	1.52 ± 0.47 (n = 7)	0.95 ± 0.18 (n = 45)	0.57

For the cytostatic drugs (chlorambucil, mafosfamide, fludarabine, cladribine, vincristine, doxorubicin or methylprednisolone) log_10 _LC_90 _concentrations were compared. In case of ionizing γ-irradiation with a fixed dose of 2 Gy, percentages of surviving cells were compared. p53 WT: wild type p53 gene. Mean +/- SEM is given. Statistical significance was calculated by means of the Kruskal-Wallis test.
